# Peripheral Nerve Blocks for Cesarean Delivery Analgesia: A Narrative Review

**DOI:** 10.3390/medicina59111951

**Published:** 2023-11-04

**Authors:** Lisa Sangkum, Amornrat Tangjitbampenbun, Theerawat Chalacheewa, Kristin Brennan, Henry Liu

**Affiliations:** 1 Department of Anesthesiology, Faculty of Medicine, Ramathibodi Hospital, Mahidol University, Bangkok 10400, Thailand; lisa.sangkum@gmail.com (L.S.); mae_ra131@hotmail.com (A.T.); drtheerawat@gmail.com (T.C.); 2 Department of Anesthesiology, Penn Medicine Lancaster General Hospital, 555 N Duke St., Lancaster, PA 17602, USA; kristin.brennan@pennmedicine.upenn.edu; 3 Department of Anesthesiology and Critical Care, Perelman School of Medicine, The University of Pennsylvania, 3400 Spruce Street, Philadelphia, PA 19104, USA

**Keywords:** cesarean section, analgesia, perioperative care, peripheral nerve block, neuraxial analgesia

## Abstract

Effective postoperative analgesia using multimodal approach improves maternal and neonatal outcomes after cesarean delivery. The use of neuraxial approach (local anesthetic and opioids) and intravenous adjunctive drugs, such as nonsteroidal anti-inflammatory drugs and acetaminophen, currently represents the standard regimen for post-cesarean delivery analgesia. Peripheral nerve blocks may be considered in patients who are unable to receive neuraxial techniques; these blocks may also be used as a rescue technique in selected patients. This review discusses the relevant anatomy, current evidence, and advantages and disadvantages of the various peripheral nerve block techniques. Further research is warranted to compare the analgesic efficacy of these techniques, especially newer blocks (e.g., quadratus lumborum blocks and erector spinae plane blocks). Moreover, future studies should determine the safety profile of these blocks (e.g., fascial plane blocks) in the obstetric population because of its increased susceptibility to local anesthetic toxicity.

## 1. Introduction

Cesarean delivery is the most commonly performed abdominal surgery in the world, and it can be associated with severe postoperative pain [1]. Optimal pain control is a fundamental pillar for better outcomes and optimal recovery. Poor postoperative pain control may delay functional recovery, impair mother–baby interaction, complicate breastfeeding [2], and increase the risk of persistent postsurgical pain [3]. Current practices for post-cesarean delivery analgesia include neuraxial opioids and/or local anesthetics and nonopioid analgesics, such as nonsteroidal anti-inflammatory drugs and acetaminophen [4,5]. Optimal analgesia after cesarean delivery involves several key considerations. First, opioid-related adverse effects should be avoided to enhance postoperative recovery. Second, the potential transfer of analgesic drugs into breast milk should be well aware off. For instance, opioids, especially through the intravenous route, can potentially transfer into breast milk and cause neonatal sedative effects. Therefore, opioid-sparing analgesia is highly recommended for this specific patient population. Finally, early mobilization is also recommended to reduce the risk of thrombotic events as well as to enhance the mother’s ability to care for her newborn baby.

In non-obstetric surgical settings, peripheral nerve block techniques have been shown to provide excellent analgesia, decrease opioid usage, enhance the quality of recovery, and reduce hospital stay [6,7,8]. Although peripheral nerve blocks are not routinely performed as a standard regimen of postoperative analgesia in cesarean delivery, these blocks may be useful as a part of multimodal analgesia in patients in whom neuraxial morphine is contraindicated and in patients with severe breakthrough pain during the postoperative period.

This narrative review provides an overview of the relevant anatomy for cesarean section, options for peripheral nerve blocks for cesarean delivery, and the advantages and disadvantages of various peripheral nerve block techniques.

## 2. Anatomy and Innervation of the Uterus and Related Structures

The lower anterolateral abdominal wall has four muscles: the rectus abdominis, external oblique, internal oblique, and transversus abdominis muscles. All the muscles, cutaneous tissue, and skin are mainly supplied by thoracoabdominal, iliohypogastric, and ilioinguinal nerves [9]. The thoracoabdominal nerves are derived from the anterior rami of the thoracic spinal nerves (T6–T12). At the mid-axillary line, they each give off a branch called the lateral cutaneous branch of the spinal nerve, which supplies the lateral abdominal wall. The iliohypogastric and ilioinguinal nerves are the terminal branches of the anterior ramus of the L1 spinal nerve. However, there is significant variability, and these nerves may originate from the T12 or L2/L3 nerve roots [10,11]; these nerves emerge from the lateral aspect of the psoas major muscle and run inferolaterally on the ventral surface of the transversus abdominis and quadratus lumborum muscles. The iliohypogastric nerve is larger and runs cephalad to the ilioinguinal nerve. Both nerves then run anterior to the quadratus lumborum (QL) muscles and leave the transverse abdominis plane compartment near the anterior superior iliac spine [9].

**Figure 1:**
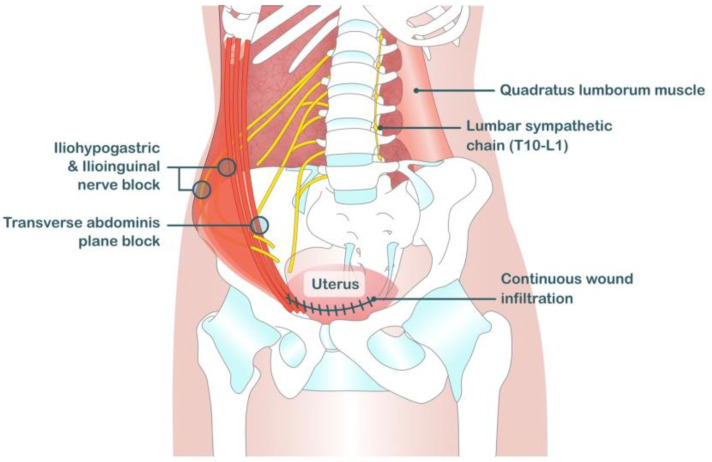
Anatomy and innervation of uterus and related structures.

The internal pelvic organs are innervated by the autonomic, sympathetic, and parasympathetic nervous systems. However, the hypogastric plexus (T10–L1) is the main autonomic nervous system of the pelvis [12] (Figure 1).

### 2.1. Peripheral Nerve Blocks for Cesarean Section

As a part of enhanced recovery after cesarean delivery, especially when neuraxial analgesic approach is suitable or contraindicated, a peripheral nerve block can play an important role in postoperative pain management cesarean after cesarean section. It improves analgesia and minimizes postoperative opioid requirements. Moreover, it may be an alternative rescue strategy when other modalities have failed or for patients with severe acute pain [9]. There are several techniques for peripheral nerve blocks.

### 2.2. Wound Infiltration

In cesarean delivery, the analgesic duration of a single-shot infiltration is limited to 4–12 h [10,11]. Therefore, continuous wound infusion may be preferred over the single-shot technique. A meta-analysis indicated that, among patients undergoing cesarean delivery, both single-shot and continuous wound-infusion techniques decreased opioid consumption by −9.69 mg [95% confidence interval (CI): −14.85 to −4.52], and they had a minimal effect on pain scores, with a mean difference in visual analog scale [VAS] scores of −0.36 [−0.58 to −0.14]) [13]. However, the analgesia benefit of either technique is very limited in patients receiving neuraxial opioid [14].

Many studies have analyzed the use of single-shot wound infiltration for post-cesarean delivery pain control. In a study of patients who did not receive intrathecal morphine, wound infiltration of 0.25% bupivacaine 40 mL with 5 µg/mL adrenaline decreased opioid consumption in the first 12 h compared with the placebo group (19 vs. 24 mg, *p* ≤ 0.001), whereas opioid consumption in the first 24 h was comparable between the groups [15]. Other studies have also observed a comparable analgesic efficacy of single-shot wound infiltration with a transversus abdominis plane (TAP) block in patients who did not receive neuraxial morphine with the TAP block group [16,17].

Continuous wound infiltration provides an extended duration of analgesia compared to single-shot wound infiltration. The local anesthetic directly inhibits noxious impulses from the site of injury and may inhibit visceral nociceptive input. Ranta et al. [18] observed that, compared with patients undergoing subfascial wound infusion, those receiving epidural infusion had a significantly lower pain score in the first 4 h (NRS at test: 1.8 vs. 3, *p* = 0.006) but not at other time points. Moreover, the 72 h postoperative opioid consumption was comparable between the groups (rescue oral oxycodone: 32 vs. 37 mg, *p* = 0.6). Kainu et al. [19] compared 0.16 mg intrathecal morphine and above-the-fascia wound infusion with 0.375% ropivacaine 5 mL/h, finding that more rescue oxycodone was required in the wound-infusion group (oxycodone consumption: 48 ± 23 vs. 26 ± 21 mg, *p* = 0.004). Another study compared subcutaneous wound infusion with and without ketorolac in patients receiving intrathecal morphine and concluded that additional ketorolac was associated with improved postoperative pain and reduced opioid consumption [20]. A randomized controlled trial by Lalmand et al. revealed that compared with the placebo group, 0.1 mg intrathecal morphine and sub-fascial wound infusion prolonged the time to the first oral opioid and reduced morphine consumption. Moreover, the two treatments had comparable analgesic effects and similar side-effect profiles [21].

Regarding catheter placement, the insertion of the catheter below the fascia of the abdominal muscle and the peritoneum results in better efficacy, as demonstrated in colorectal surgery [22] and cesarean delivery [23]. This is probably because of less leakage and the anti-inflammatory effect of local anesthetic. Both the fascia of the abdominal muscles and the peritoneum are richly innervated tissue, contributing to postoperative pain and mechanical hyperalgesia [24]. However, the optimal agents, dose of local anesthesia, and infusion regimen (e.g., continuous infusion, intermittent bolus) remain indeterminate.

The current evidence does not seem to indicate the superiority of wound infiltration or infusion techniques over neuraxial opioid administration. However, local anesthetic wound-infiltration techniques remain valuable analgesic options in patients who cannot receive intrathecal morphine or who undergo cesarean section under general anesthesia. These techniques have well-documented beneficial effects in reducing postoperative pain and opioid requirements and may have improved maternal satisfaction with pain management. Local anesthetic wound infiltration or infusion is efficacious, safe, and easy to perform. Furthermore, subfascial continuous wound infusion is preferable to single infiltration, and the use of liposomal bupivacaine infiltration in cesarean delivery requires further evaluation.

### 2.3. Liposomal Bupivacaine

Liposomal bupivacaine is a prolonged-release formulation of conventional bupivacaine. However, the current evidence of its analgesic efficacy is still questionable. A 2021 systematic review and meta-analysis comparing the analgesic efficacy of liposomal and plain bupivacaine in local anesthesia, infiltration, and regional anesthesia in abdominal, hip, knee, and hand surgery showed a small beneficial effect on pain score and opioid consumption, with a mean 24 h pain score difference of −0.37 (95%CI −0.56 to −0.19) and mean difference in 24 h morphine equivalents of 0.85 (95%CI 0.82 to 0.89) when compared between both drug formulations [25] and a 24 h opioid-sparing effect. This limited analgesic efficacy was also found in another systematic review and meta-analysis study that compared the clinical effectiveness of liposomal and plain conventional bupivacaine for peripheral nerve blocks. The results showed a nonclinical significance in the area under the curve of 24 to 72 h pain scores by 1.0 cm · h (95% I 0.5 to 1.6; *p* = 0.003) [26].

Regarding the study of liposomal bupivacaine in cesarean delivery, the first study on this subject was a single-center retrospective study [27]. They compared the analgesic efficacy of a bilateral single-shot TAP block of 10 mL liposomal bupivacaine (133 mg) admixed with 15 mL 0.25% plain bupivacaine and a control group in patients who received IT morphine 100 mcg. The liposomal bupivacaine group had a reduced morphine requirement and mean area under the curve of pain scores. However, Habib et al. reported that, compared with intrathecal morphine 150 mcg, the liposomal bupivacaine TAP block group had a greater pain score and opioid requirement [28].

Regarding the wound infiltration technique, Prabhu et al. compared the analgesic efficacy between patients who received local wound infiltration of liposomal bupivacaine and a placebo group. Both groups of patients received neuraxial morphine and were scheduled 30 mg of ketorolac every 6 h for 24 h after delivery. The results showed that the pain score (median 48 h pain score (IQR): 4 (2, 5) vs. 3.5 (2, 5.5), *p* = 0.72) and morphine requirement (median 48 h morphine equivalents: 37.5 (7.5, 60) vs. 37.5 (15, 75), *p* = 0.44) were comparable between groups [29]. Therefore, the use of liposomal bupivacaine in cesarean delivery is still limited, and further investigations are required.

### 2.4. Ilioinguinal/Iliohypogastric Blocks

The iliohypogastric nerve provides sensory innervation to the skin in the inguinal region. The ilioinguinal nerve provides sensory innervation to the skin of the labia majora and medial thigh. Iliohypogastric and ilioinguinal nerve blocks can be provided under landmark- or ultrasound-guided techniques.

In a study that included women who did not receive neuraxial opioids, those who received bilateral landmark ilioinguinal/iliohypogastric blocks had better pain scores and lower postoperative analgesic requirements than the control group [30]. Although some studies have indicated the analgesic efficacy of TAP and ilioinguinal/iliohypogastric blocks in cesarean delivery, others have reported inconsistent results [31,32]. A recent meta-analysis revealed that both approaches had similar postoperative analgesic efficacy following cesarean delivery; thus, either could be selected as an opioid-sparing technique [33].

Whether additional ilioinguinal/iliohypogastric blocks are effective after the administration of intrathecal morphine post-cesarean delivery remains controversial. Wolfson et al. reported that multiple-injection ilioinguinal/iliohypogastric nerve blocks with the landmark technique provided better pain scores and decreased postoperative opioid requirements compared with intrathecal morphine. In contrast, Vallejo et al. concluded that adding bilateral ultrasound-guided ilioinguinal/iliohypogastric blocks to intrathecal morphine administration did not improve the analgesic effect [34]. Adding ilioinguinal/iliohypogastric nerve blocks to TAP blocks may enhance analgesic effects in patients receiving intrathecal morphine and multimodal analgesia [35]. Both nerves ascend to pierce the internal oblique muscle and lie on the plane between the internal and external oblique muscle aponeurosis (not in the TAP plane) at the inferomedial to anterior superior iliac spine. Therefore, the results of ilioinguinal and iliohypogastric nerve blocks, whether by landmark or ultrasound guidance, are inconsistent.

In conclusion, ilioinguinal–iliohypogastric nerve blocks can provide some analgesic benefits in patients who did not receive neuraxial opioids and have some opioid-sparing effects. Ilioinguinal–iliohypogastric nerve blocks may be used in patients who underwent cesarean section under general anesthesia or as an alternative rescue strategy where other modalities have failed.

### 2.5. TAP Block

**Figure 2:**
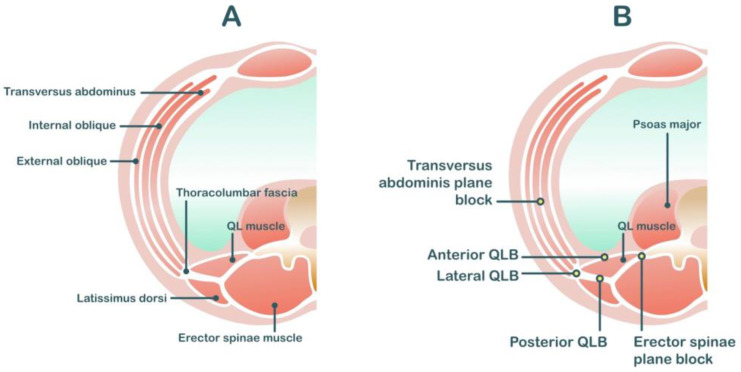
Anatomy related to transversus abdominis plane block (**A**) and quadratus lumborum block (**B**).

The TAP block is a field block between the transversus abdominis and internal oblique muscles, which contains the thoracolumbar nerve T7–L1 [29] (Figure 2). Therefore, it provides only incisional analgesia and does not affect visceral pain from the uterus. There are three main approaches to TAP blocks [36]. First, the subcostal approach is described as the deposition of local anesthetic at the TAP compartment in the anterior abdominal wall (beneath the costal margin) [37]. This approach is suitable for upper abdominal surgeries (e.g., open cholecystectomy). Second, the lateral approach is described as the deposition of local anesthetic at the TAP compartment in the lateral abdominal wall between the mid- and anterior axillary lines. Finally, the posterior approach targets the TAP compartment at the anterolateral aspect of the QL muscle or at the level of the triangle of Petit. Both of the latter techniques are suitable for lower abdominal surgeries (e.g., cesarean delivery and total abdominal hysterectomy).

The point of injection plays a critical role in local anesthetic spreading. A posterior approach to the TAP block is preferable. It provides more local anesthetic spreading to the paravertebral space and, therefore, provides better analgesic efficacy than the lateral approach [38]. The typical duration of sensory blockade in TAP block was reported to be up to 12 h, with a mean analgesic effect of 9.5 h (interquartile range: 8.5 to 11.9) [39]. For patients who require longer analgesia durations, a catheter-based technique may be preferable.

A first-of-its-kind trial compared analgesic efficacy between TAP block and control groups in cesarean delivery patients. Neither group received neuraxial morphine, but all the patients received rectal diclofenac and acetaminophen at the end of surgery. The TAP block group had a lower pain score (median VAS (IQR): 0.5 (0, 1) vs. 2 (1, 4)) and required less morphine consumption (mean morphine consumption (SD): 18 ± 14 vs. 66 ± 26 mg, *p* < 0.001) compared with the control group. Multiple randomized controlled studies, including posterior or lateral TAP block to multimodal analgesia, have indicated that TAP block had analgesic benefits and opioid-sparing effects compared with the placebo group [40,41,42,43]. Systematic reviews and meta-analyses have evaluated the use of TAP and QL blocks for post-cesarean delivery analgesia. El-Boghdadly et al. reviewed the efficacy of TAP and QL blocks after cesarean delivery with the primary outcomes of cumulative 24 h morphine-equivalent consumption. In women who did not receive intrathecal morphine, TAP block reduced opioid consumption with a mean difference of 21.9 mg (95% CI 12.17 to 31.61). However, in patients receiving intrathecal morphine, no difference in analgesic efficacy was noted between the TAP block and intrathecal opioid groups, with a mean difference of −2.10 (95% CI −10.21 to 6.01) [44].

Local anesthetic systemic toxicity has been reported after TAP blocks in cesarean delivery patients [45,46]. Griffith et al. studied the level of plasma concentration in patients who received ultrasound-guided TAP block after wound closure (2.5 mg/kg of ropivacaine diluted to 40 mL). The results showed that 12 out of 30 patients had total plasma ropivacaine concentrations that exceeded the quoted toxic threshold (concentration of 2.2 mcg/mL) at some time after the block [47]. The obstetric population is susceptible to local anesthetic toxicity because their nerve axons become more sensitive as cardiac output increases and protein binding decreases [48], thus necessitating a minimally effective dose of local anesthetic for this population. Ng et al. conducted a meta-analysis comparing the analgesic efficacy between a high dose (>50 mg per block side) and a low dose (≤50 mg per block side) of TAP block. Compared with the control groups, the high-dose and low-dose TAP block groups had lower 24 h morphine-equivalent consumption with a mean difference of −22.41 mg (95% CI −38.56, −6.26) and −16.29 mg (95%CI −29.74, −2.84), respectively. There were no differences between the high- and low-dose groups in terms of opioid consumption, time to first analgesia, or 24 h pain scores. The results indicated that there was no difference in analgesic efficacy [49]. Because the TAP block is a fascial plane block, the volume of local anesthetic may affect the adequate local anesthetic spreading. However, the minimal effective volume remains inconclusive. Lower-concentration, higher-volume strategies (with local anesthetic volume ≥15 mL/side) were recommended in a meta-analysis conducted by Abdallah et al. This finding is consistent with that of a cadaveric study that reported that a greater volume of 15 mL provided more extensive spreading than a lower volume [50].

In conclusion, TAP blocks are effective but do not confer additional analgesia when neuraxial morphine is included. The posterior TAP approach is usually preferred over the lateral TAP approach. The TAP block may be considered an opioid-sparing technique in women who underwent cesarean section under neuraxial or general anesthesia but did not receive intrathecal opioids. The TAP block may provide a rescue technique in patients with moderate-to-severe postoperative pain after cesarean delivery.

### 2.6. Quadratus Lumborum Blocks (QL Block)

The QL block is an interfascial plane block like the TAP block but has the potential for more diffuse analgesia. This is because of the injection of a local anesthetic into the thoracolumbar fascia (TLF), which connects with the back muscle and lumbar paravertebral region. The local anesthetic injected adjacent to the QL muscle and posterior to the transversalis fascia may spread to the thoracic paravertebral space along the TLF to block the somatic nerves, which are posterior to the arcuate ligaments of the diaphragm, and the lower level of the thoracic sympathetic trunk [51].

Because the QL block involves a more posterior approach than the TAP block, local anesthetic solution can spread into the paravertebral space. Therefore, the QL block potentially covers analgesia in both somatic and visceral pain and theoretically provides better analgesia than TAP blocks [52]. There are three common approaches to QL block: the lateral approach, where local anesthetics are injected lateral to the QL muscle; the posterior approach, where the local anesthetics is injected posterior to the QL muscle; and the anterior approach, where the local anesthetics are injected into the plane between the QL and psoas major muscles (Figure 2). The dermatome coverage provided by the QL block depends on the approach, varying from T6 to L4 [53,54,55].

The first randomized controlled trial in cesarean delivery was conducted by Blanco et al. in 2015. None of the patients received neuraxial morphine or multimodal analgesia. The results showed that the QL block group had a better VAS pain score (median 24 h VAS (IOR) 2 (0, 3) vs. 4 (2, 5), *p* = 0.006) and required less morphine consumption (median morphine consumption (IOR) 11 (4, 18) vs. 19 (11, 36), *p* = 0.011) compared with the control group. Subsequent studies have reported QL blocks to be superior to control groups [56,57]. A current meta-analysis by Zhao et al. indicated that QL blocks provided greater analgesia and reduced postoperative opioid requirements (24 h mean difference, −11.51 mg; 95% CI −17.05 to −5.96) in patients who did not receive intrathecal morphine. In addition, the time to first analgesic requirement and the incidence of postoperative nausea and vomiting were also significantly reduced by QL block [58].

Comparing neuraxial morphine with QL block, Tamura et al. found that patients receiving spinal morphine (100 mcg) had lower VAS scores and 24 h morphine requirements than patients receiving QL block. Several randomized controlled trial studies have also reported greater analgesic efficacy of neuraxial morphine over QL block [59,60]. Irwin et al. found that patients receiving intrathecal morphine (0.1 mg) and multimodal analgesia (rectal diclofenac 100 mg and intravenous acetaminophen 1 gm) had comparable 24 h morphine consumption in the QL and sham block groups (median (IQR) 12 mg (8, 29) vs. 14 mg (5, 25)) and 24 h VAS (median (IQR) 18 (2, 30) vs. 19 (3, 25)). A systematic review and meta-analysis study in 2021 by Hussian et al. suggested that QL block does not enhance analgesic outcomes whether combined with or without spinal morphine. The mean differences in 24 h opioid consumption and VAS score at 4 to 6 h for spinal morphine and spinal morphine combined with QL block were 0 mg (−2 to 1) and −0.1 cm (−0.7 to 0.4), respectively. For spinal morphine and QL block, the differences were 7 mg (−2 to 15) and 0.6 cm (−0.7 to 1.8), respectively. Therefore, QL block was found to not improve analgesic outcomes in patients who received intrathecal morphine [61].

Local anesthetic systemic toxicity remains a risk with interfascial plane block. A large dose and volume of local anesthetic is required. A local anesthetic plasma concentration study showed that the peak concentration of local anesthetics is lower after QL block than after TAP block [62]. Moreover, the potential risks may include hematoma from bleeding because of the presence of lumbar arteries, which are located at the posterior and lateral aspects of the QL muscle. Lower-limb weakness and hypotension have also been reported after QL block because anesthetics can spread to the lumbar plexus [63] and the paravertebral space [64]. Kadoya et al. compared quadricep power by using a dynamometer between patients who received anterior QL block and a control group. The incidence of quadriceps weakness was reported to be up to 30% in the QL group [65]. Therefore, these adverse effects should be considered in patients receiving QL block.

Taken together, these findings indicate that QL block can provide analgesic benefits for patients in whom neuraxial morphine is contraindicated. However, QL blocks did not provide additional benefits to those who received neuraxial opioids. Therefore, QL block is indicated for patients who did not receive neuraxial morphine or received other analgesic strategies that failed.

### 2.7. Erector Spinae Plane Blocks

**Figure 3:**
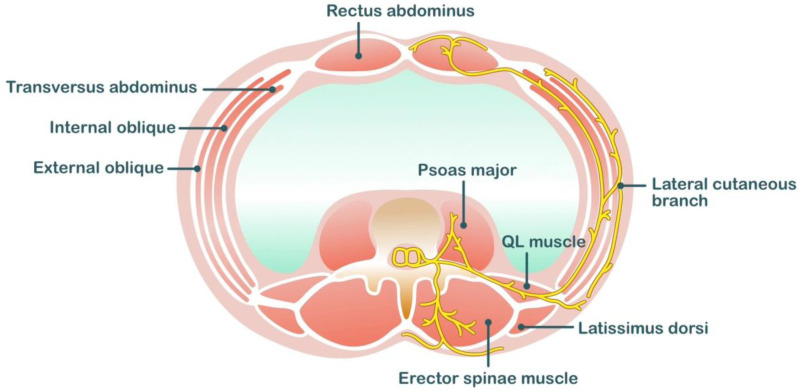
Anatomical related to erector spinae plane block.

Local anesthetic is deposited in the interfascial plane between the erector spinae muscle and the tips of the vertebral transverse processes in the erector spinae plane (ESP). Local anesthetics spread within this potential space in the craniocaudal direction. A study on the spread of local anesthetics after ESP block indicated that the distribution of one dermatome level requires 3.4 mL of local anesthetics [66]. Eventually, the local anesthetics spread to the paravertebral space, where they can act on the ventral rami and spinal nerve roots. The ventral rami (intercostal nerves) provide sensory innervation to the anterolateral wall, and the dorsal ramus provides sensory innervation to the posterior wall (Figure 3).

In patients who did not receive intrathecal opioids, bilateral ESP block at the T9 level could decrease 24 h postoperative fentanyl consumption (279 ± 242.99 mg vs. 423.08 ± 212.55 mg, *p* = 0.003) and prolong time to first analgesic requirement (150.20 ± 51.83 min vs. 197.60 ± 84.49 min, *p* = 0.022) [67]. Two randomized controlled trials compared the analgesic efficacy of ESP block and TAP block. Both studies showed that ESP provided better analgesic efficacy and more prolonged analgesic effects than TAP block [68,69]. Hamed et al. conducted a randomized controlled trial to evaluate the analgesic effects of ESP block versus 100 mcg of intrathecal morphine. The ESP block group required less tramadol consumption for up to 48 h (101.71 ± 25.67 mg vs. 44 ± 16.71 mg, *p* < 0.001) and had a longer time to first analgesic (4.93 ± 0.82 h vs. 12 ± 2.81 h) than the intrathecal morphine group [70]. A systematic review and meta-analysis study in 2022 investigating the efficacy of ESP block for cesarean delivery included only three articles and concluded that ESP block decreased the total tramadol consumption but did not decrease the postoperative pain score [71]. In addition, one case report described the potential risk of motor block in a patient who received ESP blocks [72].

In conclusion, ESP block is relatively new in the obstetric population and warrants further research to elucidate its analgesic efficacy and safety profile.

## 3. Summary

Multimodal analgesia is an effective pain-management strategy for cesarean delivery. Long-acting neuraxial opioids (e.g., morphine, diamorphine) with adjunct drugs, such as acetaminophen and nonsteroidal anti-inflammatory drugs, are currently the standard regimen for cesarean delivery in most parts of the world. Peripheral nerve blocks are considered an alternative for patients for whom neuraxial opioids are not suitable and are used as a rescue technique in some patients, though future applications of these peripheral nerve blocks may expand significantly. The possible advantages and disadvantages of each regional anesthetic technique are summarized in Table 1. Most recent articles have lacked the statistical power to determine whether any peripheral nerve block technique is superior to neuraxial morphine as well as their safety and cost-effectiveness. Therefore, further studies are required to delineate the analgesic efficacy in specific patient groups of each individual regional anesthetic technique, especially with newer blocks (e.g., QL block and ESP block), and evaluate the safety profile of the various blocks, particularly fascial plane blocks.

## Figures and Tables

**Table 1:** Comparison efficacy of analgesia regarding regional anesthetic techniques.

Peripheral Nerve Block Techniques	Dermatomal Coverage	Somatic Analgesia	Visceral Analgesia	Potential Risks	Analgesic Efficacy
Wound infiltration technique Single shot Continuous infusion (CI)	Variable	+	-	Leakage around the wound Catheter dislodgement (for CI)	Single-shot wound-infiltration technique limits their duration of action 4–12 h [10,11] Subfascial continuous wound infusion is recommended. [22,23] Neither technique is superior over neuraxial opioid administration. [13]
Liposomal bupivacaine	Variable	+	-	Local anesthetic systemic toxicity	Duration was reported to be up to 72 h. Evidence on liposomal bupivacaine in clinical use is still limited.
Ilioinguinal/iliohypogastric blocks (II-IH blocks)	T12-L1	+	-	Intra-arterial injection	Duration was reported to be up to 24 h. II-IH blocks provide analgesic benefits in patients who did not receive neuraxial opioids and have some opioid-sparing effects. [33]
Transversus abdominis plane block (TAP block)	T10-L1	+	-	Local anesthetic systemic toxicity Hematoma Internal organ injury	Duration was reported to be up to 6–12 h [39] TAP blocks are effective but do not confer additional analgesia when neuraxial morphine is included. [44] The posterior TAP approach is preferred over the lateral TAP approach. [38]
Quadratus lumborum block (QL block) Lateral approach Posterior approach Anterior approach	Depends on the approach, varying from T6 to L4	+	Possibly provides visceral analgesia	Local anesthetic systemic toxicity Hematoma Hypotension [63] Internal organ injury Quadriceps motor block [64]	Duration was reported to be up to 24–48 h. QL block can provide analgesic benefits in patients in whom neuraxial morphine is contraindicated. QL blocks did not provide additional benefits in those who received neuraxial opioids.
Erector spinae plane block	Variable	+	Possibly provides visceral analgesia	Local anesthetic systemic toxicity Possible quadriceps motor block [72]	Duration was reported to be up to 24 h. Only 4 RCTs currently available.

## Data Availability

primary data available upon request.

## Abbreviations

CI	Confidence interval
ESP	Erector spinae plane
QL	Quadratus lumborum
TAP	Transversus abdominis plane
VAS	Visual analog scale
